# Analgesic efficacy of local versus proximal nerve blocks after hallux valgus surgery: a systematic review

**DOI:** 10.1186/s13047-022-00581-0

**Published:** 2022-10-22

**Authors:** Hamid Reza Ravanbod

**Affiliations:** Division of Podiatric Medicine and Surgery, University Western Australia, Park Avenue Building, Crawley Ave, WA 6009 Crawley, Australia

**Keywords:** Bunionectomy, Hallux valgus, Nerve block, Analgesic effect, Postoperative pain

## Abstract

**Background:**

Hallux valgus (HV) surgery is an orthopaedic procedure that commonly causes mild to moderate postoperative pain. Effective management of this pain has become an important element of modern hallux valgus surgical treatment. A local anaesthetic (LA) with an antinociceptive effect can control this pain. However, relatively few papers have evaluated this strategy in depth. The objective of the current systematic review was to address this demand by comparing the efficacy of proximal and local blocks in controlling postoperative pain following hallux valgus surgery.

**Main text:**

Ovid-MEDLINE, Cochrane Central, PubMed, Web of Science (WOS), Scopus, and Embase were searched from their inceptions through December 29, 2021. Observational and clinical trial publications in peer-reviewed English-language journals with a sample size of at least 20 were included. The trials involved adults over 18 who could describe their discomfort and had a bunionectomy. The included studies were evaluated using the Cochrane risk of bias 2 method. Descriptive analysis synthesised the results.

Among the 439 articles identified, five studies compromising 459 participants were included. Ankle blocks were superior to control in two studies (*P* = 0.001, *P* < 0.001) and superior to local blocks in one study (*P* < 0.001). Additionally, one study showed that popliteal and ankle blocks administered with lidocaine or levobupivacaine were equivalent (*P* = 0.123 and *P* = 0.055, respectively). However, one of these five included studies indicated that ankle blocks were not effective (*P* = 0.123) in reducing postoperative pain.

**Conclusions:**

The key findings presented herein suggest that regional blocks effectively reduce postoperative pain and that an ankle block has more supportive evidence for its effectiveness. However, an adequate assessment of the effectiveness of various administrative routes was challenging due to the lack of reliable evidence. This needs to be addressed in future studies.

**Trial registration:**

PROSPERO registration: CRD42022307974.

**Supplementary Information:**

The online version contains supplementary material available at 10.1186/s13047-022-00581-0.

## Background

### Rationale

Hallux valgus (HV) or bunion is a progressive deformity of the first ray of the foot that causes lateral displacement of the proximal phalanx on the metatarsal head [1]. The overall prevalence of bunion in the adult population is 23% [2]. In Australia, 82.3% of bunions are found in females and 8.50% are in those ≥ 45 years old [2]. Australians see a GP for bunions 4.2% per 10,000 encounters [3].

Conservative methods can be used to control HV, but they do not repair the deformity. Surgery offers a potentially permanent solution [1]. Corrective surgery for HV is a common elective operation. In 2007, bunion surgery was the second most prevalent performed operation (20.8%) among Australian podiatric surgeon graduates [4].

Different studies have employed bunion surgery and preoperative injections to measure analgesic effectiveness. Most bunionectomy patients are healthy, but they have chronic, severe pain [5]. Bunions require soft tissue and bone surgeries [6]. Due to the good health of patients, studying analgesic effects in bunionectomy may be done with little confounding from participant health, and results can be expanded to many other procedures due to their parallels with bunionectomy [7]. Preoperative injections can block peripheral nerve inputs early after surgery and avoid central hyperexcitability, which can amplify local anaesthetic (LA) effects [8]. Central sensitisation increases the excitability of medullary and spinal dorsal horn neurones through a cascade of events. This phenomenon happens due to the nerve or tissue injury irrespective to the general anaesthesia and causes progressively worsening pain after surgery [8]. Increases in nociceptor activity can lead to long-term hyperexcitability in the nervous system and amplification of pain, hence preoperative regional blocks in surgeries such as bunionectomy can block this activity in peripheral nociceptors and yield better outcomes [8].

This evaluation used local anaesthetics (LAs) to give regional blocks. LAs decrease neuronal excitation and conduction by raising depolarisation thresholds [9]. Lidocaine and levobupivacaine are LAs typically used for regional blocks. Regional blocks for bunion surgeries in this review included popliteal blocks, ankle blocks and local blocks (i.e., Mayo and peri-incisional blocks) [9]. Popliteal blocks target the sciatic nerve prior to its branching into the common peroneal and tibial nerves. An ankle block targets the posterior tibial, saphenous, superficial peroneal, deep peroneal, and sural nerves. Local nerve blocks, such as Mayo and peri-incisional, anaesthetize the nerve branches around the first ray [10].

Regardless of surgical approach, postoperative (bunionectomy) pain is common, hence localised blocks are important. Local anaesthetic (LA), opioids, or N-methyl-D-aspartate (NMDA) receptor antagonists are utilised to block afferent pathways and disrupt pathological pain cycles [11]. In order to prevent opioid dependency and other undesirable consequences, recent focus has shifted from opioid to non-opioid analgesic regimes [11]. Administering a regional LA block is a non-opioid approach to decrease bunionectomy pain.

### Objectives

The current systematic review aimed to compare and assess the analgesic efficacy of the proximal (popliteal and ankle) blocks and local (Mayo and peri-incisional) blocks in patients undergoing HV procedures. The objective was to establish recommendations for the management of acute postoperative pain in patients with HV.

## Methods

### Protocol/registration

The protocol was registered with PROSPERO (registration number: CRD42022307974). This quantitative systematic review followed the Preferred Reporting Items for Systematic Reviews and Meta-Analyses (PRISMA) [12] (Additional file 1:Appendix 1) standards (National Institute for Health and Care Research, n.d.).

### Strategy and sources

The databases were searched from their inceptions till December 29, 2021. Missed studies were found using Google Scholar. Article reference lists were also searched. Search terms were: Analgesic effect, anaesthetic, ankle block, popliteal block, Mayo block, peri-incisional block, bunion, hallux valgus, pain treatment, postoperative pain. These phrases and synonyms were combined using Boolean operators ('AND,' 'OR,' and '*'). When the full text was unavailable or there was missing data, associated authors were contacted to discover another research. Additional file 1: Appendices 2A-F include the database search strategy.

### Eligibility criteria

Articles were selected using the following criteria: English-language peer-reviewed observational studies and clinical trials having at least 20 participants. The included trials involved adults over 18 who could describe their discomfort and had a bunionectomy. Entries up to December 2021 was included. In this evaluation, pure LAs were injected preoperatively to reduce pain. Mayo and peri-incisional blocks were local; ankle and popliteal blocks were proximal. Pain treatment strategies that combined analgesics or corticosteroids with LAs, continuous infiltration or recurring bolus infiltration of LAs, and post-surgical anaesthetic injections were eliminated. Measurement techniques were used to categorise experiments for synthesis. The included trials examined pain using a visual analogue scale (VAS) ranging from 0 to 10 cm or 0 to 100 mm, where 0 indicated no pain and 10 or 100 indicated the most severe pain [13]. In the included trials, 0 represented no pain and 10 represented severe pain [14].

### Data selection and collection

EndNote X9 (Thomson Reuters, Carlsbad, CA, USA) was used to filter titles and abstracts [15] and remove duplicates. Each record was examined using the study criteria. This systematic review used full-text publications. Each stage was evaluated by the author.

The reviewer constructed a Microsoft Excel table to gather the following data from the papers: Name, country, patient demographics (mean age and gender), research design, intervention and comparator group features, LA injection techniques, continuous or bolus injection, preoperative or postoperative injection, LA dose, LA injection route, and tourniquet appliers (GA). Patient main characteristics, patterns of LA administration for postoperative pain, mean change in pain scores on VAS or NRS, percentage of pain-free participants, time to first rescue analgesia, time to mobilisation, pethidine consumption, time to first rescue analgesic, tramadol consumption, number of PCA bolus demands, nausea, and vomiting were also studied. If data from a trial was available at multiple times throughout each preceding period, all data was retrieved and compared. WebPlotDigitizer was used to extract data from graphs [16]. Table data was used throughout the review.

### Quality assessment

The studies were appraised using the revised Cochrane risk of bias 2 methodology [17]. One person assessed quality. RoB 2 includes randomisation bias, intervention variations, missing outcome data, outcome measurement, and outcome selection. The same methodology was applied to each study, separately. Synthesis was carried out using narrative assessment findings. Overall risk of bias was defined as the greatest risk of bias in any of the investigated domains (Table 1 and Fig. 4).

**Table 1:** RoB 2 in the included studies

Study	Randomisation process	Deviation from the intervention	Missing outcome data	Measurement of the outcome	Selection of the reported result	Overall
Gadek et al., (2015) [18]	L	C	L	L	C	C
Su et al., (2019) [19]	H	C	L	H	H	H
Migues et al., (2005) [20]	C	H	L	H	C	H
Turan et al., (2007) [21]	L	H	L	H	H	H
Özhan et al., (2020) [22]	L	C	L	L	C	C

*C* Some concerns, *H* High risk, *L* Low risk, *RoB2* Risk of bias tool 2

### Synthesis

Inferential data analysis was not appropriate given the heterogeneity of treatments, locations, research designs, and outcome measures [23]. The author utilised descriptive analysis with a quantitative methodology to generate tables and graphs. The studies were categorised by their outcome measuring tools and individually assessed.

The primary analysis included continuous variables such as the proportion of patients with no postoperative discomfort or the average VAS or NRS score. The author used the STATA automation tool [24] to build forest plots between intervention and control. Due to study variations, comparing forest plot from different study results was not possible.

## Results

### Study selection

**Fig. 1:**
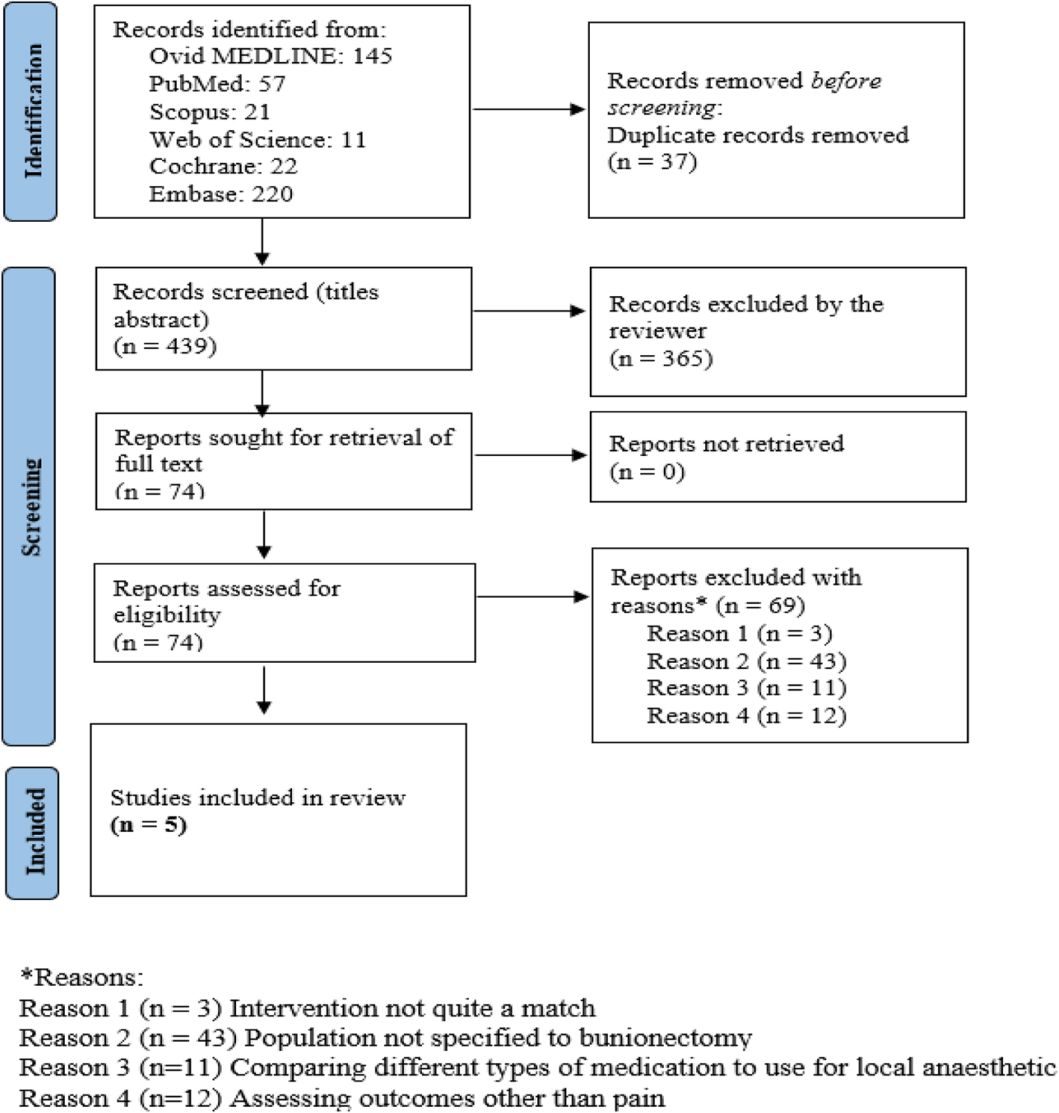
PRISMA flow diagram

Databases produced 476 studies. 37 duplicate studies were eliminated. 365 papers were removed after reading titles and abstracts, and 74 full-text articles were included. 69 papers were excluded due to non-matching interventional groups (3 studies), no demographic matching (43 studies), comparison of different LA medications (11 studies), or non-pain outcomes (12 studies). Finally, five articles fit the review [18–22, 25] (Additional file 1: Appendices 3A-E) Fig. 1 depicts the PRISMA flow diagram for this systematic review. Additional file 1: Appendix 4 contains citations and reasoning for articles rejected during full-text screening.

### Study characteristics

All relevant studies were hospital-based randomised trials (Table 2). Two studies administered ankle blocks [21, 22], one article explored local blocks [18], one research compared local blocks to ankle blocks [19], and one study compared the popliteal block to ankle blocks [20]. None of the final publications on local blocks addressed Mayo (Additional file 1: Appendices 3A—B).

**Table 2:** Characteristics of each study

Study	Population size	Duration of study (months)	Female (%)	Age (years) (mean ± SD)	Follow-up duration (POH)	Study design	Funding source	Intervention 1	Intervention 2	Control	Measurement tool	Outcome for postoperative pain
Gadek et al., (2015) [18]	118	32	84%	47.8	24	Prospective patient blind randomised trial	The author	Local block	NR	Normal saline	VAS	Effective
Su et al., (2019) [19]	90	NR	23 on 1	20–65	36	Prospective patient blind randomised trial	NR	Local block	Ankle block	Without the regional anaesthetic	NRS	The ankle block better than the local block better than the control
Migues et al., (2005) [20]	51	4–8	NR	61 ± 11	NR	Randomised prospective	NR	Ankle block	Popliteal block	NR	VAS	Both were equal
Turan et al., (2007) [21]	90	NR	NR	45 ± 15	24	Randomised patient blind	Associate Professor I. Turan	Ankle block	NR	Saline	VAS	Not effective
Özhan et al., (2020) [22]	110	NR	92.7%	60.5 ± 9.4	12	Prospective patient blind randomised trial	Private Çankaya Hospital	Ankle block	NR	Without the regional anaesthetic	VAS	Effective

*NR* Not reported, *NRS* Numerical rating scale, *POH* Postoperative hours, *VAS* Visual analogue scale

### Risk of bias within studies

**Fig. 2:**
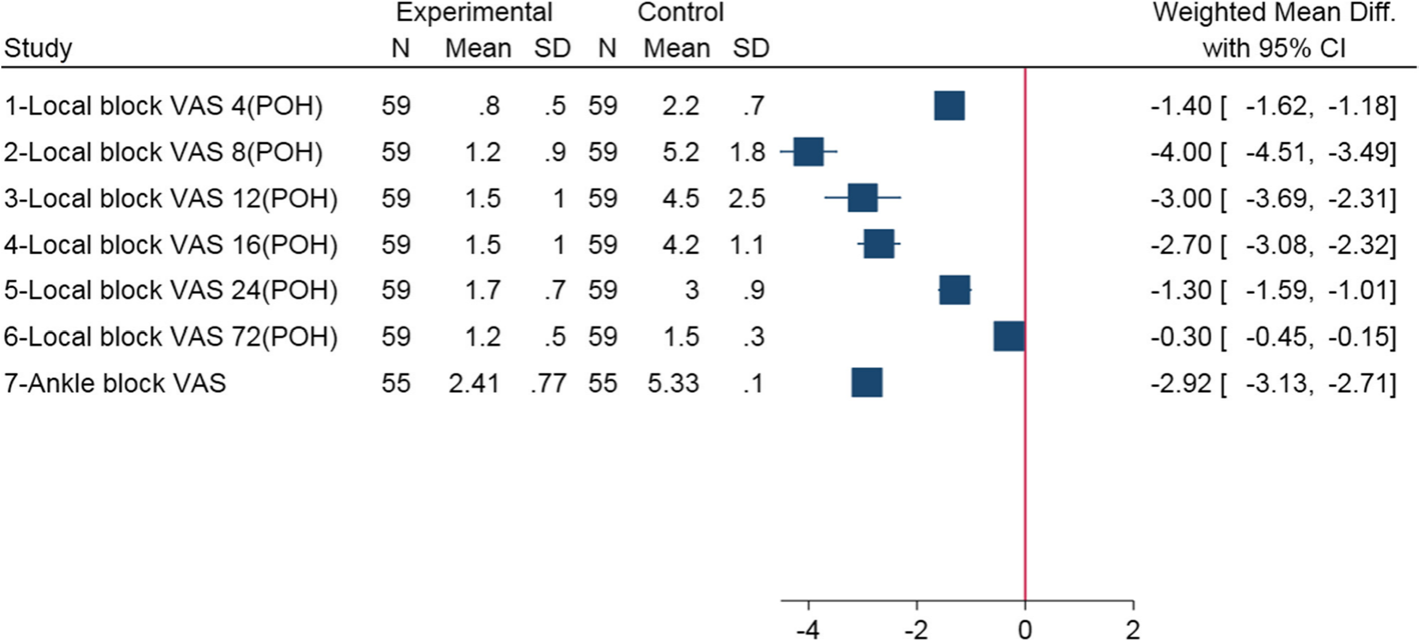
The weighted mean differences in VAS reports from local and ankle blocks. CI: Confidence Interval; N: Number; POH: Postoperative hours; SD: Standard deviation

The Cochrane library's RoB 2 tool was used to identify bias in each listed study. Figures 2 and 4 summarise the investigations. Although all five articles were randomised, only three showed sufficient randomisation quality. Five studies adequately handled missing outcome data. Other RoB 2 elements, including intervention deviation, outcome measurement, and result selection, showed a high risk.

### Results of individual studies

In total, five studies involving 459 cases ranging in age from 20 to 72 years were studied in this review. Among the cases, at least 80% were female. Only one study had no control [20]. Three studies had a sample size of greater than 90 participants, Su et al. [19], included 75 participants, whereas Migues et al., included 51 participants. Further details regarding these studies are available in Additional file 1: Appendices 2A—E.

### Results of syntheses

#### Similarities and differences among the included studies

 As well as commonalities based on Population, Intervention, Comparator, and Outcome (PICO), Table 3 shows non-PICO similarities among the included studies. The same table also provides examples of the disparities present.

**Table 3:** Examples of the similarities and disparities among the studies according to various criteria

Characteristics	Studies
**Similarities**	
**Female was the dominant sex**	All studies
**Type of study was randomised trial**	All studies
**Tibial and common peroneal nerves blocked during the ankle block**	All studies where ankle blocks were administered ankle blocks
**L**A **was a combination of lidocaine 2% and levobupivacaine 0.5%**	All studies except the Su et al. (2019) [19]
**Administered oral/IV GA**	All studies except for the Gadek et al. (2015) [18]
**Participants were classified 1–3 according to American anaesthetics staging (AAS)**	All studies except for the Gadek et al. (2015) [18] and Migues et al. (2005) [20]
**Disparities**	
**Administered spinal anaesthesia**	Gadek et al. (2015) [18]
**Discussed size of bunion preoperatively**	Gadek et al. (2015) [18]
**Discussed type of bunionectomy**	Gadek et al. (2015) [18]
**Administered midazolam and fentanyl injections before the ankle block**	Özhan et al. (2020) [22]
**Added sural nerve blocks to the ankle block**	Turan et al. (2007) [21]
**Added saphenous nerve blocks to the ankle block**	Özhan et al. (2007) [22] and Migues et al. (2005) [20]
**Excluded patients with neurologic and cognitive disorders**	Özhan et al. (2007) [22] and Su et al. (2019) [19]
**Utilized** u**ltrasound for administering the ankle block**	Özhan et al. (2007) [22] and Su et al. (2019) [19]
**Block was administered before GA**	Özhan et al. (2007) [22]
**The anaesthetist administered the regional block**	Özhan et al. (2007) [22]
**The orthopaedic surgeon administered the regional block**	Turan et al. (2007) [21]

*AAS* American anaesthetics staging, *IV* Intra venous, *GA* General anaesthesia, *LA* Local anaesthesia

According to Table 3, the included studies had similarities that allowed an analysis and comparison [20]. However, there were heterogeneities in the population demographics, intervention design and extent of reporting. Due to these heterogeneities, it was impossible to conduct an inferential analysis; therefore, a descriptive analysis was used for this review [20].

#### Results from VAS measurement

As shown in Table 4, Gadek et al. [18] reported that a local block had a significant effect (*P* < 0.001) in the first 24 POH with a maximum WMD = -4 VAS (95% CI: -4.51, -3.49) in the 8 POH. A study by Özhan et al. [22] concluded that an ankle block was also considered effective in the same group (*P* = 0.001) (Tale 4).

**Table 4:** VAS results in local block

**Study**	**Time from tourniquet release (POH)**	**Groups**		***P-value***
		**Intervention (mean ± SD)**	**Control (mean ± SD)**	
Gadek et al., 2015 [18] (A peri-incisional block versus placebo)	4	0.8 ± 0.5	2.2 ± 0.7	< 0.001
	8	1.2 ± 0.9	5.2 ± 1.8	< 0.001
	12	1.5 ± 1.0	4.5 ± 2.5	< 0.001
	16	1.7 ± 0.7	4.2 ± 1.1	< 0.001
	24	1.3 ± 1.1	3.0 ± 0.9	< 0.001
	72	1.2 ± 0.5	1.5 ± 0.3	> 0.05
Özhan et al., 2020 [22] (An ankle block versus no block)	NR	5.33 ± 1.1	2.41 ± 0.77	0.001

*POH* Postoperative hours; *NR* Not reported, *VAS* Visual analogue scale

The forest plot shown in Fig. 2 illustrates the effectiveness of the peri-incisional and ankle blocks in controlling postoperative pain using the WMD. Local (peri-incisional) blocks were most effective 8 POH (WMD = -4, 95% CI: -4.51, -3.49). Ankle block was also effective (WMD = -2.92, 95% CI: -3.13, -2.71). Analysis and quantification of differences between variables were completed within each research study. However, given the heterogeneity of the studies included in this review, pooled analysis was not practical.

#### Results from pain free reports

Studies by Turan et al. [21] and Migues et al. [20] presented the percentage of patients who reported being pain-free or had a VAS score of 0. Turan et al. [21] evaluated the pain experienced following ankle block with lidocaine, levobupivacaine, or a placebo (saline, 15 mL). This study divided the postoperative period into several periods, as shown in Table 5.

**Table 5:** Percentage of people who reported being pain-free after an ankle block

Measuring tool	Pain-free percentage							
Location	**Ankle blocks**							
Medication	**Lidocaine**				**Levobupivacaine**			
Timing	ADS	EDS	MPD	APD	ADS	EDS	MPD	APD
Intervention group (Pain free percentage)	40	45	32	25	49	43	50	42
Control group (Pain free percentage)	47	42	52	45	47	42	52	45
*P-value*	0.123	0.123	0.123	0.123	0.052	0.052	0.052	0.052

*APD* Afternoon postoperative day 1, *ADS* Afternoon day of surgery, *EDS* Evening day of surgery, *MPD* Morning postoperative day 1

The study by Turan et al. [21] found that regardless of the type of LA employed in this trial, ankle blocks could not alleviate postoperative pain (*P* ≥ 0.052). Additionally, Migues et al. [20] examined the pain-free percentage after ankle and popliteal blocks and concluded that they were equivalent (*P* = 0.123). However, this study did not include a control, and the impact of each block was not quantifiable, as shown in Table 6.

**Table 6:** Percentage of pain-free reports following the ankle and popliteal blocks

Location	Ankle blocks				Popliteal blocks				*P-value*
Timing (POH)	6	12	18	24	6	12	18	24	
Intervention (Pain free percentage)	58	30	38	39	70	45	40	42	0.123
Control	NR								

*NR* Not reported, *POH* Postoperative hours

#### Results from NRS measurement

Su et al.’s [19] study compared the control (no block), local (peri-incisional) and ankle blocks in the NRS group. Postoperative pain in this study was divided into activity and rest pain. Activity pain was defined as pain during ankle movement. The authors assessed postoperative pain at 6, 24 and 36 POH. According to the authors, ankle (*P* < 0.001) and peri-incisional (*P* = 0.026) blocks were both effective at reducing postoperative activity and rest pain after the first six POH, although the ankle block was more effective (*P* < 0.001), as shown in Table 7.

**Table 7:** NRS results from ankle and local blocks in terms of rest and activity pain

Time interval (POH)	NRS pain reports (mean ± SD)						P-value*	
	**Control**		**Local blocks**		**Ankle blocks**			
	**Rest**	**Activity**	**Rest**	**Activity**	**Rest**	**Activity**	**Rest**	**Activity**
**6**	2.3 ± 1.0	3.2 ± 2.6	1.8 ± 0.8	1.3 ± 2.0	0.2 ± 0.4	0.4 ± 1.0	≤ 0.01	< 0.001
**12**	1.2 ± 1.2	2.6 ± 1.8	1.3 ± 1.0	1.5 ± 1.4	1.8 ± 0.4	2.2 ± 2.0	NR	0.086
**24**	0.7 ± 0.8	1.7 ± 1.5	0.6 ± 0.6	1.9 ± 1.9	0.5 ± 0.4	1.3 ± 1.4		0.437
**36**	0.2 ± 0.2	0.9 ± 1.0	0.6 ± 1.6	1.0 ± 1.3	0.2 ± 0.2	0.8 ± 1.0		0.868

*NR* Not reported, *NRS* Numerical rating scale, *POH* Postoperative hours, *SD* Standard deviation

^*^P-values were obtained using ANOVA test

**Fig. 3:**
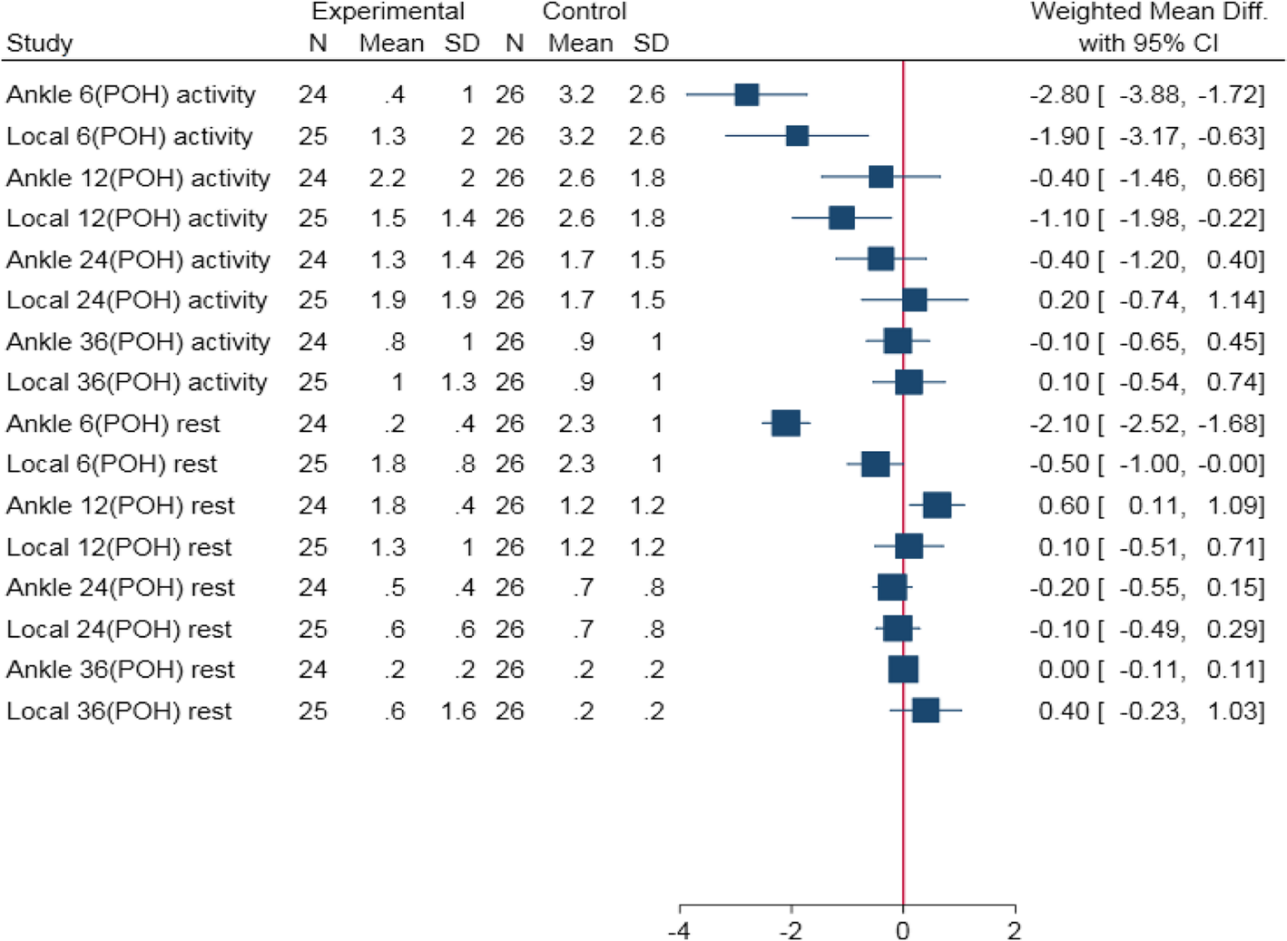
The weighted mean differences in NRS reports during activity and rest. CI: Confidence Interval; POH: Postoperative hours

The forest plot in Fig. 3 indicates that ankle blocks had a substantially greater effect throughout the first six POH both for active (WMD = -2.80, 95% CI: -3.88, -1.72) and rest pain (WMD = -2.10, 95% CI: -2.52, -1.68).

#### Secondary outcome

Other manifestations of pain in patients were discussed in the Özhan et al. [22] and Su et al. [19] studies. For instance, Su et al. [19], quantified pain that interferes with sleep or mood reported it using an NRS ranging from 0 to 10, where 0 indicated no sleep or mood disturbance and 10 indicated the maximum level of sleep or mood disturbance. Meanwhile, the dosage of injected rescue analgesic (fentanyl) was significantly lower in the ankle block group than in other two groups (*P* < 0.001) (Table 8).

**Table 8:** Secondary outcomes for postoperative pain (Sleep and mood)

Time interval (POH)	Control (mean ± SD)			Peri-incisional block (mean ± SD)			Ankle block (mean ± SD)			P-value*		
	**Sleep** **(NRS)**	**Mood** **(NRS)**	**Fentanyl dosage (mg)**	**Sleep** **(NRS)**	**Mood** **(NRS)**	**Fentanyl dosage**	**Sleep** **(NRS)**	**Mood** **(NRS)**	**Fentanyl dosage (mg)**	**Sleep**	**Mood**	**Fentanyl**
**6**	1.4 ± 2.2	1.4 ± 2.1	170 ± 40	0.9 ± 0.2	0.4 ± 1.4	100 ± 40	0.4 ± 1.4	0.1 ± 0.6	20 ± 2.1	0.231	0.012	< 0.001
**12**	0.5 ± 1.3	0.6 ± 1.3	110 ± 39	0.4 ± 0.9	0.4 ± 1.4	60 ± 38	0.8 ± 1.5	0.1 ± 0.2	10 ± 3.7	0.430	0.225	< 0.001
**24**	0.3 ± 0.5	0.1 ± 0.5	140 ± 42	0.1 ± 0.6	0.4 ± 1.2	59 ± 44	0.2 ± 0.4	0.2 ± 0.6	70 ± 5.2	0.585	0.496	< 0.01
**36**	0.1 ± 0.2	0.0 ± 0.2	80 ± 21	0.1 ± 0.4	0.0 ± 0.4	40 ± 44	0.1 ± 0.6	0.0 ± 0.0	30 ± 2.4	0.797	0.531	< 0.001

*POH* Postoperative hours, *SD* Standard deviation

^*^P-values were obtained using ANOVA test

Özhan et al. [22] discussed the type, dose and duration of rescue analgesics given to patients and the average time to mobilisation following surgery. The frequency of patients experiencing nausea and vomiting was also recorded in this study, as shown in Table 9.

**Table 9:** Secondary outcomes for postoperative pain (various manifestations)

Outcome	Time to first rescue analgesic (POH) (mean ± SD)	Time to mobilisation (POH) (mean ± SD)	Pethidine consumption (mg) (mean ± SD)	Pethidine required (n, %)	Time to first rescue analgesic (POH) (mean ± SD)	Tramadol consumption via PCA (mg) (mean ± SD)	Number of PCA bolus demands (n) (mean ± SD)	Nausea and vomiting (n, %)
Intervention	5.9 ± 2.27	2.1 ± 0.25	2.27 ± 0.61	3 (5.5%)	5.9 ± 2.27	80.6 ± 7.3	4.1 ± 0.7	1 (1.8%)
Control	1.32 ± 0.92	4.11 ± 2.25	11.27 ± 10.35	17 (30.9%)	1.32 ± 0.92	113.8 ± 10.2	2.2 ± 3.4	11 (20.4%)
*P- value*	0.001	0.001	0.001	0.001	0.001	0.002	0.002	0.002

*POH* Postoperative hours, *mg* milligrams, *PCA* Patient-controlled analgesia, *SD* Standard deviation

The authors of these studies employed the secondary outcome results to determine the severity of pain. While these markers might help better understand postoperative pain in patients, they did not focus on pain at the surgical site which makes their use for this review problematic [25].

### Risk of bias across studies

**Fig. 4:**
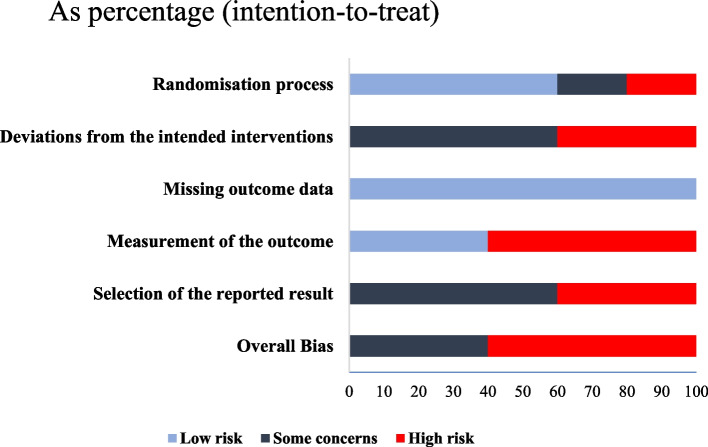
The risk of bias among all the included studies

Although computing cumulative evidence was not practical in this descriptive analysis, a summary bar chart was created to analyse the overall risk (Fig. 4). The chart indicated that the overall risk level in this study was quite high (Fig. 4).

## Discussion

### Summary of evidence

The current systematic review compared the analgesic efficacy of proximal (popliteal and ankle) to local (Mayo and peri-incisional) blocks in HV surgery patients. This analysis included five trials assessing regional block effectiveness, with 459 individuals aged 25 to 72. Regarding demographic characteristics of the included studies, the study by Su et al. [19] had the smallest sample size (24 participants) in one of its two intervention groups, while Gadek et al. [18] had the largest population size, with 118 participants (59 in each group). There was a higher proportion of females than males in three of the five included studies (Table 2), while the other two studies also reported this higher proportion in their intervention groups (Additional file 1: Appendices 2C—D). The lowest and highest proportions of females to males were 24:6 and 29:1, respectively, both of which belonged to the intervention groups of Turan et al. [21]. This study also included the youngest population (mean age; 47.0 ± 13.3 years old) [21], whereas the Migues et al. [20] study contained the oldest age population (61.0 ± 11.0 years old). The key findings of the present review were that for postoperative pain management blocking peripheral nerves using LA was more effective than not blocking at all. In bunionectomy patients, ankle blocks were equivalent to popliteal blocks and better than peri-incisional blocks. Some of the included trials showed that an ankle block reduced mood problems during the first six POH, the need for rescue analgesics, and postoperative nausea/vomiting. The next paragraphs discuss these findings.

In HV surgical patients, ankle blocks were more effective than local blocks or a control (no blocks) [19, 22]. An ankle block before GA was more effective than a placebo, according to Özhan et al. [22]. In this study, postoperative pain was assessed for 12 POH, and an ankle block decreased pain (WMD: -2.92 VAS, 95% CI: -3.13, -2.71). Su et al. [19] compared ankle nerve blocks to peri-incisional blocks and controls. Activity and rest discomfort NRS ratings were recorded at 6, 12, 24, and 36 POH. The study reported that pre-treatment ankle nerve blocks were better to peri-incisional blocks and control up to six POH. Even though two of the five investigations corroborated this effectiveness [19, 22], Turan et al. [21] showed that an ankle block did not significantly reduce postoperative pain compared to saline (Additional file 1: Appendix 3D). However, the authors still found that an ankle block reduced the requirement for intraoperative GA. A reduction in GA demand intraoperatively tends to be better systemically for the patient in the long run [11], thus although Turan et al. [21] did not conclude that an ankle block played a major influence postoperatively, it did intraoperatively. Consequently, it could be asserted that ankle blocks provide greater postoperative pain management than peri-incisional blocks after bunionectomies.

The only research to explore the popliteal block, found that it was equally effective as an ankle block in treating postoperative pain. Both groups were tracked for 24 POH. The lack of a control group in this study makes it impossible to compare these results with those from other investigations [26]. Small study size (51 individuals), older age cohort [26], and simultaneous foot operations potentially affect the accuracy of this evaluation [13].

Local (peri-incisional) blocks were assessed as an alternate strategy for controlling postoperative pain after HV correction surgery. Su et al. [19] found a local block to be more effective than none. Local (peri-incisional) blocks reduced activity and rest pain up to 12 POH, according to research [2], and at six POH had maximal impact (WMD: -1.90 NRS, 95%, CI: -3.17, -0.63). This research still favoured ankle blocks for postoperative pain management. Gadek et al. [18] only evaluated peri-incisional blocks with the longest follow-up time (72 POH). Their investigation showed peri incisional blocks are better than controls. Local (peri-incisional) block was effective for eight POH (WMD: -4 VAS, 95% CI: -4.51, -3.49). It should be noted that this was the only study that administered a spinal block instead of GA. Therefore, both investigations found peri-incisional blocks useful for pain management following bunionectomy, with Su et al. [19] showing ankle blocks were more effective.

Özhan et al. [22] and Su et al. [19] had less important but still meaningful results. These have been categorised as secondary outcomes in this review (Tables 8 and 9). Özhan et al. [22] found that ankle nerve block combined GA reduced the demand for pethidine and tramadol compared to GA alone. In the intervention group, the time to first rescue analgesic usage and mobility improved. Ankle block reduces nausea and vomiting. Notably, trauma, stress, and postoperative immobilisation can also produce similar symptoms [27]. Su et al. [19] showed considerably less mood disturbance in ankle block patients compared to peri-incisional block patients and control groups after six POH, but no significant changes thereafter. No significant difference in disrupted sleep after surgery was seen between the three groups. Ankle blocks lowered postoperative fentanyl usage and patient demand during the first 36 POH. Overall, both trials supported regional blocks, especially ankle blocks, for controlling postoperative pain following bunionectomy.

Randomization in included trials reduces probable confounders, yet it can still contribute to systematic review heterogeneity and confounding [28]. For instance, people with higher BMIs may feel greater discomfort [29], as well as red-haired patients, as they are harder to anaesthetize [30]. These variables have not been discussed in part of the included studies properly; however, they can still play a confounding role. Other heterogeneities among the research include gender, age, anaesthetic drugs and procedures, procedure choice and applied fixation, follow-up period, and bunion severity before operation. These parameters were sometimes studied, although the findings differed. For example, the studies have different female and age proportions. Generally, bunions are more prevalent among females over the age of 18 [1, 31], and bunionectomy in individuals below 18 is uncommon [32]. However, despite these overall trends being present in the higher proportion of females than males and age ranges in participants across all samples, the proportions varied significantly among the included studies, suggesting that they may still have played a confounding role [28]. Gadek et al. used spinal anaesthesia instead of oral or IV GA [18]. In other research, individuals who had spinal anaesthesia instead of GA reported less discomfort and problems [33]. Gadek et al. [18] reported intrathecally injecting 12.5 mg of bupivacaine 5 mg/ml. Spinal anaesthesia can considerably lessen postoperative pain. The mechanism of action and duration depend on the fat and protein content of the administered medication, which regulates its nervous system penetration [34].

Unlike popliteal block, spinal block inhibits the sciatic nerve and autonomic nervous system more proximally and produces more side effects and patient dissatisfaction [35].

Therefore, in the literature, spinal anaesthesia was utilised largely for anaesthetic purposes and its postoperative analgesic effects were not examined [36].

A spinal block in Gadek et al. 's investigation [18] may have skewed the systematic review's findings [33]. Different experiments employed different analgesics and dosages. For instance, Özhan et al. [22] injected midazolam and fentanyl before an ankle block and Pethidine (0.5 mg/kg) was also given IV as a rescue analgesic. In the trial by Gadek et al. [18], patients received IV ketoprofen (100 mg) and paracetamol (1000 mg) three times after surgery, commencing at four POH, with 7.5 mg of morphine sulphate as a rescue analgesic. Su et al. [19] reported that patients received fentanyl through PCA a few hours postoperatively at 1 g/kg and 0.33 g/kg. Block analgesic effectiveness may have been affected by the analgesic activity and half-life of several medications. This can bias trials with and without analgesics [25]. Follow-up should be appropriate for the half-life of the LAs utilised; otherwise, changes in LA efficacy may confound regional block results [26]. The timing of LAs before or after GA [37] or tourniquet administration [31], expertise level of the surgeon or person providing the block [38], and kind of bunionectomy procedure [39] might also matter. Among the articles included in this study, only Gadek et al. [18] explains the type of bunion surgery utilized (Table 3), which is the chevron osteotomy. However, over a hundred distinct bunionectomy procedures have been recorded [40]. The choice between these operations depends on the degree of the deformity and surgeon's preferences. Most patients will have a corrective osteotomy, with or without an additional soft tissue procedures (Fig. 2). In Australia, both the chevron and scarf osteotomies are regularly performed. Mild to moderate malformations are often treated with a distal metatarsal osteotomy, however severe deformities may necessitate a proximal osteotomy or tarsometatarsal joint fusion. In cases of severe arthritis (hallux rigidus), a fusion of the MTPJ may be recommended. Simple excision of the bony prominence (bunionectomy) is uncommon due to the high likelihood of recurrence and the dissatisfaction of patients with the outcome [40]. Infrequently are soft tissue operations performed alone; they are more often employed as an adjuvant to osteotomy [40]. Additionally, the use of minimally invasive surgical procedures is on the rise, although most studies have found no change in patient function, complication rates, or clinical outcomes when compared to conventional approaches [41].

Among these different surgical procedures, only the percutaneous chevron/Akin osteotomy has debatable reduction in the postoperative pain level [42–45]. A study of 25 articles on hallux valgus surgery found that the surgical methods had no clinical impact on gait, quality of life, or patient satisfaction [46]. Even the type of internal fixations (bioabsorbable magnesium vs titanium screws) did not make difference in the level of postoperative pain [47, 48].

Additionally, extended tourniquet periods at high pressures result in increased pain, opioid usage, and hospital stay. It is probable that putting tourniquet pressures on limb occlusion pressure (LOP), will increase the safety margin of tourniquets [49].

Overall, different variables may remain hidden in the included studies while skewing the findings of the systematic review [50]. Therefore, included research should address these issues as much as feasible.

### Clinical relevance

This review's results will benefit patients by reducing their postoperative pain. This knowledge will also benefit surgeons, anaesthetists, nurses, and rehabilitation practitioners [11].

### Limitations

This is the first systematic review to investigate the effects of preoperative LA on postoperative pain in HV surgery, however bias and limitations still exist.

In terms of bias, Gadek et al. [18] and Ozhan et al. [22] pose some concerns. The reviewer also believes three other research papers [19–21] raise high risk. Gadek et al. [18] and Ozhan et al. [22] had intervention discrepancies (where planned patients declined to participate in the study and insufficient information regarding the protocol was available). Also, neither Turan et al. [21] nor Migues et al. [20] were double-blind. This raises the likelihood of measuring errors or intervention deviation [28]. These studies didn't mention their chosen analytic strategy, which might affect outcome selection. Su et al. [19] also faced risks, including deviating from the planned intervention (it is unclear if participants knew about it), outcome measurement (no information on the assessor), and result selection (the person performing the intervention was not blinded to the project). Overall, these hazards affect the review's dependability.

Regarding limitations, it is to be noted that the current review has limitations arising from two sources. Firstly, in this field there is a scarcity of relevant papers to study, with only five deemed to meet the inclusion criteria for the current review. According to the study by Pannucci et al., this is due to the difficulties associated with surgical randomisation, blinding, patient variability or control, and this is a significant drawback [51]. Secondly, there were limitations in the five studies on which the current review is based. There were heterogeneity factors among the included studies which precluded any inferential analysis [52]. In a descriptive analysis, the efficacy was determined by assessing the frequency of supportive interventions, though this frequency cannot be accurately assigned because it may be attributable to factors other than the intervention [28]. Given the relatively small sample size in part of included studies and the high-risk levels, our findings should be interpreted with caution [53].

Nevertheless, the present review is a valuable contribution to bunionectomy pain reduction and has transferability to comparable surgical procedures. It also offers advice for future low-risk investigations.

## Conclusion

After assessing the literature, the author supports the principle of administration of regional nerve blocks, preferably an ankle block, as an effective modality for the management of postoperative pain in bunionectomy. Administering such blocks are supported by the current literature given that a regional nerve block administration for a bunionectomy procedure may contribute to postoperative pain reduction. However, given the present heterogeneity, high risk of bias, and confounding factors involved in the studies included in this review, it is additionally recommended that further randomised control trials with larger sample sizes be conducted to confirm these conclusions.

## Supplementary Information

**Additional file 1.**

## Data Availability

Not applicable.

## Abbreviations

ADS: Afternoon day of surgery
APD: Afternoon postoperative day 1
CI: Confidence Interval
GA: General Anaesthetic
LA: Local Anaesthetic
LOP: Limb occlusion pressure
mg: Milligram
μg/kg: Microgram to kilogram
mL: Millilitre
MPD: Morning post day surgery
NIHR: National Institute for Health and Care Research
NMDA: N-methyl-D-aspartate
NR: Not reported
NRS: Numerical rating scale
PCA: Patient-controlled analgesia
PICO: Population, Intervention, Comparator, Outcome
POH: Postoperative hours
PRISMA: Preferred Reporting Items for Systematic Reviews and Meta-Analyses
PROSPERO: Prospective Register of Systematic Reviews
RoB: Risk of bias
SD: Standard Deviation
UWA: The University of Western Australia
VAS: Visual Analogue Scale
Weighted mean diff.: Weighted mean difference
WMD: Weighted mean difference
WOS: Web of Science
