# Supplementary material for: Analgesic efficacy of local versus proximal nerve blocks after hallux valgus surgery: a systematic review
Source: J Foot Ankle Res. 2022 Oct 22;15:78. doi: 10.1186/s13047-022-00581-0 (PMC9588208; doi:10.1186/s13047-022-00581-0)
Supplement: Supplementary file 1 — Additional file 1. [file 13047_2022_581_MOESM1_ESM.pdf]

1. Appendices

Appendix 1 PRISMA 2020 Checklist

Section and Topic	Item #	Checklist item	Location where item is reported
**TITLE**			
Title	1	Identify the report as a systematic review.	i
**ABSTRACT**			iii
Abstract	2	See the PRISMA 2020 for Abstracts checklist.	iii
**INTRODUCTION**			1
Rationale	3	Describe the rationale for the review in the context of existing knowledge.	1
Objectives	4	Provide an explicit statement of the objective(s) or question(s) the review addresses.	2
**METHODS**			3
Eligibility criteria	5	Specify the inclusion and exclusion criteria for the review and how studies were grouped for the syntheses.	3
Information sources	6	Specify all databases, registers, websites, organisations, reference lists and other sources searched or consulted to identify studies. Specify the date when each source was last searched or consulted.	3
Search strategy	7	Present the full search strategies for all databases, registers and websites, including any filters and limits used.	3
Selection process	8	Specify the methods used to decide whether a study met the inclusion criteria of the review, including how many reviewers screened each record and each report retrieved, whether they worked independently, and if applicable, details of automation tools used in the process.	4
Data collection process	9	Specify the methods used to collect data from reports, including how many reviewers collected data from each report, whether they worked independently, any processes for obtaining or confirming data from study investigators, and if applicable, details of automation tools used in the process.	4
Data items	10a	List and define all outcomes for which data were sought. Specify whether all results that were compatible with each outcome domain in each study were sought (e.g., for all measures, time points, analyses), and if not, the methods used to decide which results to collect.	4
	10b	List and define all other variables for which data were sought (e.g., participant and intervention characteristics, funding sources). Describe any assumptions made about any missing or unclear	4

Section and Topic	Item #	Checklist item	Location where item is reported
		information.	
Study risk of bias assessment	11	Specify the methods used to assess risk of bias in the included studies, including details of the tool(s) used, how many reviewers assessed each study and whether they worked independently, and if applicable, details of automation tools used in the process.	5
Effect measures	12	Specify for each outcome the effect measure(s) (e.g., risk ratio, mean difference) used in the synthesis or presentation of results.	5
Synthesis methods	13a	Describe the processes used to decide which studies were eligible for each synthesis (e.g., tabulating the study intervention characteristics and comparing against the planned groups for each synthesis (item #5)).	5
	13b	Describe any methods required to prepare the data for presentation or synthesis, such as handling of missing summary statistics, or data conversions.	5
	13c	Describe any methods used to tabulate or visually display results of individual studies and syntheses.	5
	13d	Describe any methods used to synthesize results and provide a rationale for the choice(s). If meta-analysis was performed, describe the model(s), method(s) to identify the presence and extent of statistical heterogeneity, and software package(s) used.	5
	13e	Describe any methods used to explore possible causes of heterogeneity among study results (e.g., subgroup analysis, meta-regression).	5
	13f	Describe any sensitivity analyses conducted to assess robustness of the synthesized results.	5
Reporting bias assessment	14	Describe any methods used to assess risk of bias due to missing results in a synthesis (arising from reporting biases).	5
Certainty assessment	15	Describe any methods used to assess certainty (or confidence) in the body of evidence for an outcome.	5
**RESULTS**			6
Study selection	16a	Describe the results of the search and selection process, from the number of records identified in the search to the number of studies included in the review, ideally using a flow diagram.	6
	16b	Cite studies that might appear to meet the inclusion criteria, but which were excluded, and explain why they were excluded.	6
Study characteristics	17	Cite each included study and present its characteristics.	6-8
Risk of bias in	18	Present assessments of risk of bias for each included study.	9

Section and Topic	Item #	Checklist item	Location where item is reported
studies			
Results of individual studies	19	For all outcomes, present, for each study: (a) summary statistics for each group (where appropriate) and (b) an effect estimates and its precision (e.g., confidence/credible interval), ideally using structured tables or plots.	9 and Appendices 2A - 2F
Results of syntheses	20a	For each synthesis, briefly summarise the characteristics and risk of bias among contributing studies.	11 and table 1 and Appendices 2A - 2F
	20b	Present results of all statistical syntheses conducted. If meta-analysis was done, present for each the summary estimate and its precision (e.g., confidence/credible interval) and measures of statistical heterogeneity. If comparing groups, describe the direction of the effect.	10-16
	20c	Present results of all investigations of possible causes of heterogeneity among study results.	10
	20d	Present results of all sensitivity analyses conducted to assess the robustness of the synthesized results.	NA
Reporting biases	21	Present assessments of risk of bias due to missing results (arising from reporting biases) for each synthesis assessed.	16
Certainty of evidence	22	Present assessments of certainty (or confidence) in the body of evidence for each outcome assessed.	NA
**DISCUSSION**			17
Discussion	23a	Provide a general interpretation of the results in the context of other evidence.	17
	23b	Discuss any limitations of the evidence included in the review.	21
	23c	Discuss any limitations of the review processes used.	21
	23d	Discuss implications of the results for practice, policy, and future research.	21
**OTHER INFORMATION**			
Registration and protocol	24a	Provide registration information for the review, including register name and registration number, or state that the review was not registered.	3
	24b	Indicate where the review protocol can be accessed, or state that a protocol was not prepared.	3
	24c	Describe and explain any amendments to information provided at registration or in the protocol.	NA
Support	25	Describe sources of financial or non-financial support for the review, and the role of the funders or sponsors in the review.	22

Section and Topic	Item #	Checklist item	Location where item is reported
Competing interests	26	Declare any competing interests of review authors.	22
Availability of data, code and other materials	27	Report which of the following are publicly available and where they can be found template data collection forms; data extracted from included studies; data used for all analyses; analytic code; any other materials used in the review.	3

Appendix 2 Search strategies

2A. Ovid-MEDLINE

#	Searches	Results
**1**	Hallux/ or Hallux Valgus/	5,267
**2**	Bunion, Tailor's/ or Bunion/	133
**3**	(Bunion* or 'Hallux abductus valgus' or 'First metatarsus varus' or hallux*).tw, kf.	6,529
**4**	or/1–3	7,843
**5**	Analgesia/ or Anaesthesia, Local/ or Nerve Block/ or Anaesthetics, Local/	81,250
**6**	(Local adj1 (infiltration or block or an? esthetic or an? esthes*)).tw, kf.	40,210
**7**	or/5–6	102,131
**8**	('proximal nerve blocks' or 'Ankle block' or 'Saphenous nerve block').tw, kw.	182
**9**	or/7–8	102,173
**11**	4 and 9	145

2B. Scopus

(Bunion* or ‘Hallux abductus valgus’ or ‘First metatarsus varus’ or hallux*) AND ((local W/1 (infiltration or block or an? esthetic or an?esthes*)) OR (‘proximal nerve blocks’ or ‘Ankle block’ or ‘Saphenous nerve block’))

2C. Web of Science:

(Bunion* or ‘Hallux abductus valgus’ or ‘First metatarsus varus’ or hallux*) AND ((local NEAR/1 (infiltration or block or an? esthetic or an?esthes*)) OR (‘proximal nerve blocks’ or ‘Ankle block’ or ‘Saphenous nerve block’))

2D. PubMed:

(Bunion* or "Hallux abductus valgus" or "First metatarsus varus" or hallux*) AND ((local AND (infiltration or block or an? esthetic or an?esthes*)) OR ("proximal nerve blocks" or "Ankle block" or "Saphenous nerve block"))

2E. Embase:

#	Searches
1	Hallux/ or Hallux Valgus/
2	Bunion, Tailor's/ or Bunion/
3	(Bunion* or 'Hallux abductus valgus' or 'First metatarsus varus' or hallux*).tw, kw
4	or/1–3
5	Analgesia/ or Anaesthesia, Local/ or Nerve Block/ or Anaesthetics, Local/
6	(Local adj1 (infiltration or block or an? esthetic or an? esthes*)).tw, kw
7	or/5–6
8	('proximal nerve blocks' or 'Ankle block' or 'Saphenous nerve block').tw, kw.
9	or/7–8
11	4 and 9

2F. Cochrane Library:

(Bunion* or "Hallux abductus valgus" or "First metatarsus varus" or hallux*) AND ((local NEAR/1 (infiltration or block or an? esthetic or an?esthes*)) OR ("proximal nerve blocks" or "Ankle block" or "Saphenous nerve block"))

Appendix 3 Characteristics of included studies.

3A. Gadek et al., (2015)

<table>
<tr><td rowspan="3">Peri-incisional block
4 ml of levobupivacaine (0.25%) plus 3 ml of lidocaine (2%)</td><td rowspan="2">Age</td><td rowspan="2">Female sex</td><td rowspan="2">BMI (Average ± SD)</td><td colspan="2">GA</td><td rowspan="2">Population size</td><td rowspan="2">Type of procedure</td><td rowspan="2">Duration of study (month)</td><td rowspan="2">Duration of measuring outcome (POH)</td><td rowspan="2">Outcomes</td><td rowspan="2">Fund Source</td><td rowspan="2">Declaration of interest</td></tr>
<tr><td>Type</td><td>Medication/ dosage</td></tr>
<tr><td>47.8</td><td>84%</td><td>22.8 ± 2.0</td><td rowspan="2">Spinal</td><td rowspan="2">Levobupivacaine (5 mg/mL, 12.5 mg)</td><td rowspan="2">59</td><td rowspan="2">Modified chevron, mini-invasive</td><td rowspan="2">32</td><td rowspan="2">24</td><td rowspan="2">VAS average</td><td rowspan="2">The author</td><td rowspan="2">Yes</td></tr>
<tr><td>Control
(7 ml saline)</td><td colspan="2">NR</td><td>22.2 ± 1.8</td></tr>
</table>

BMI: Body mass index; GA: General anaesthesia; POH: Postoperative hours; mg: milligrams; mL: millilitre; NR: Not reported; SD: Standard deviation; VAS: Visual analogue scale.

3B. Su et al, (2018)

	Age	Female sex	BMI (Average ± SD)	GA		Population size	Type of procedure	Duration of study (month)	Duration of measuring outcome (POH)	Outcomes	Fund Source	Declaration of interest
				Type	Medication/ dosage							
Ankle block 8-10 mL of levobupivacaine (0.25%)	20-65	21 on 4	23.7 ± 4.9	Oral	Lidocaine 1 mg/kg; Thiamylal 5 mg/kg; Propofol 1 mg/kg.	25	NR	NR	36	Fentanyl frequency and dose, NRS average Post op nausea/vomiting, itching	No	No
Peri-incisional 8-10 mL of levobupivacaine (0.25%)		23 on 1	22.1 ± 4.0			24						
Control (No block)	48	88.50%	23.0 ± 3.7			26						

BMI: Body mass index; GA: General anaesthesia; POH: Postoperative hours; mg/kg: milligram to kilogram; mL: millilitre; NR: Not reported; NRS: Numeric rating scale; SD: Standard deviation.

3C. Migues et al., (2005)

	Age (Average ± SD)	Female sex	BMI (Average ± SD)	GA		Population size	Type of procedure	Duration of study (month)	Duration of measuring outcome (POH)	Outcomes	Fund Source	Declaration of interest
				Type	Medication/ dosage							
Ankle block 4 mL levobupivacaine (0.5%) and 4 mL lidocaine (non epi 2%)	61 ± 11	88.5%	NR	No GA		25	Unilateral forefoot procedures	4 to 8	24	Percentage of VAS = 0	No	No
Popliteal block 2mL levobupivacaine (0.5%) and 2 mL lidocaine (Non epi 2%)	56 ± 17	92%				26						

BMI: Body mass index; GA: General anaesthesia; POH: Postoperative hours; mL: millilitre; NR: Not reported; SD: Standard deviation; VAS: Visual analogue scale.

3D. Turan et al., (2007)

	Age (Average ± SD)	Female sex	BMI (Average ± SD)	GA		Population size	Type of procedure	Duration of study (month)	Duration of measuring outcome (POH)	Outcomes	Fund Source	Declaration of interest
				Type	Medication/ dosage							
Ankle block levobupivacaine 2.5 mg/mL 15 cc	45 ± 15	24 on 6	NR	Oral	Sevoflurane, 1.03 ± 0.26	30	HV ad modum Turan and hammertoe procedure	NR	48	Need for rescue Analgesia. Pain ratings (severity of pain)	Associate Professor Ibrahim Turan	Yes
Ankle block Lidocaine 10 mg/mL 15 mL	50 ± 12	29 on 1			Sevoflurane 0.99 ± 0.35	30						
Control (15 mL saline)	46 ± 13	26 on 4			Sevoflurane, 1.43 ± 0.45	30						

BMI: Body mass index; GA: General anaesthesia; POH: Postoperative hours; mL: millilitre; mg/mL: milligrams to millilitres; NR: Not reported; SD: Standard deviation; VAS: Visual analogue scale.

3E. Özhan et al., (2020)

Ankle block	Age (Average ± SD)	Female sex	Population size	BMI (Average ± SD)	GA		Type of procedure	Duration of study (month)	Duration of measuring outcome (POH)	Outcomes	Fund Source	Declaration of interest
					Type	Medication/ dosage						
	60.5 ± 9.4	94.5%	55	NR	Oral	Propofol (2-2.5 mg/kg); Remifentanil (1mg/kg); Rocuronium (10–20mg); Fentanyl (50 µg/kg); Midazolam (3 mg)	NR	93.10 ± 18.74	12	VAS average	Private Çankaya Hospital	Yes
Control (7 mL saline)	59.76 ± 8.63	90.9%	55									

BMI: Body mass index; GA: General anaesthesia; POH: Postoperative hours; mL: millilitre; NR: Not reported; µg/kg: Microgram to kilogram; mg/kg: milligram to kilogram; SD: Standard deviation; VAS: Visual analogue scale.

Appendix 4 Reasons for exclusion of full-text reports

Reason 1 (n = 3) Intervention not quite a match

1- Wylde V, Gooberman-Hill R, Horwood J, Beswick A, Noble S, Brookes S, et al. The effect of local anaesthetic wound infiltration on chronic pain after lower limb joint replacement: A protocol for a double-blind randomised controlled trial. BMC Musculoskelet Disord. 2011 Dec;12(1):53.

2- J. EDMONDS-SEAL, M.B., B.S., F.F.A.R.C.S., GM.C. PATERSON, M.B., C.H.B., F.F.A.R.C.S. and, A.B. LOACH, M.A., M.B., B. C.H.I.R., F.F.A.C.S. Local nerve blocks for post-operative pain. Nuffield Department of Anaesthetics, Radcliffe Infirmary Oxford. 1979.

3- Casati A, Fanelli G, Koscielniak-Nielsen Z, Cappelleri G, Aldegheri G, Danelli G, et al. Using Stimulating Catheters for Continuous Sciatic Nerve Block Shortens Onset Time of Surgical Block and Minimizes Postoperative Consumption of Pain Medication After Halux Valgus Repair as Compared with Conventional Nonstimulating Catheters: Anesthesia & Analgesia. 2005 Oct;101(4):1192–7.

Reason 2 (n = 43) Population not specified to bunionectomy

1- Kamath P. A Novel Distraction Technique for Pain Management during Local Anesthesia Administration in Pediatric Patients. Journal of Clinical Pediatric Dentistry. 2013 Sep 1;38(1):45–7.

2- Remérand F, Godfroid HB, Brilhault J, Vourc'h R, Druon J, Laffon M, et al. Chronic pain 1year after foot surgery: Epidemiology and associated factors. Orthopaedics & Traumatology: Surgery & Research. 2014 Nov;100(7):767–73.

3- Emerson RH, Barrington JW, Olugbode O, Lovald S, Watson H, Ong K. Comparison of Local Infiltration Analgesia to Bupivacaine Wound Infiltration as Part of a Multimodal Pain Program. J Surg Orthop Adv. 2015;24(4):235–41.

4- Kang C, Lee GS, Kim SB, Won YG, Lee JK, Jung YS, et al. Comparison of postoperative pain control methods after bony surgery in the foot and ankle. Foot and Ankle Surgery. 2018 Dec;24(6):521–4.

5- Dang DY, McGarry SM, Melbihess EJ, Haytmanek CT, Stith AT, Griffin MJ, et al. Comparison of Single-Agent Versus 3-Additive Regional Anesthesia for Foot and Ankle Surgery. Foot Ankle Int. 2019 Oct;40(10):1195–202.

6- Murauski JD, Gonzalez KR. Peripheral Nerve Blocks for Postoperative Analgesia. AORN Journal. 2002 Jan;75(1):134–47.

7- Ma HH, Chou TFA, Tsai SW, Chen CF, Wu PK, Chen WM. The efficacy and safety of continuous versus single-injection popliteal sciatic nerve block in outpatient foot and ankle surgery: a systematic review and meta-analysis. BMC Musculoskelet Disord. 2019 Dec;20(1):441.

8- Carlock KD, Hildebrandt KR, Konda SR, Egol KA. The use of regional anaesthesia for surgical intervention has minimal effect on functional outcomes following fracture nonunion repair. Injury. 2019 Mar;50(3):671–5.

9- Li BL, Liu X, Cui L, Zhang W, Pang H, Wang M, et al. Local Infiltration Analgesia with Ropivacaine Improves Postoperative Pain Control in Ankle Fracture Patients: A Retrospective Cohort Study. Pain Research and Management. 2020 Mar 9; 2020:1–7.

10- Biddinger KR, Komenda GA, Schon LC, Myerson MS. A New Modified Technique for Harvest of Calcaneal Bone Grafts in Surgery on the Foot and Ankle. Foot Ankle Int. 1998 May;19(5):322–6.

11- Bramlett K, Onel E, Viscusi ER, Jones K. A randomized, double-blind, dose-ranging study comparing wound infiltration of DepoFoam bupivacaine, an extended-release liposomal bupivacaine, to bupivacaine HCl for postsurgical analgesia in total knee arthroplasty. The Knee. 2012 Oct;19(5):530–6.

12- Maher AJ, Metcalfe SA. A report of UK experience in 917 cases of day care foot surgery using a validated outcome tool. The Foot. 2009 Jun;19(2):101–6.

13- Joe HB, Choo HS, Yoon JS, Oh SE, Cho JH, Park YU. Adductor canal block versus femoral nerve block combined with sciatic nerve block as an anesthetic technique for hindfoot and ankle surgery: A prospective, randomized noninferiority trial. Medicine (Baltimore). 2016 Dec;95(52): e5758–e5758.

14- YaDeau JT, Goytizolo EA, Padgett DE, Liu SS, Mayman DJ, Ranawat AS, et al. Analgesia after total knee replacement: local infiltration *versus* epidural combined with a femoral nerve blockade: A prospective, randomised pragmatic trial. The Bone & Joint Journal. 2013 May;95-B (5):629–35.

15- Carli F, Clemente A, Asenjo JF, Kim DJ, Mistraletti G, Gomarasca M, et al. Analgesia and functional outcome after total knee arthroplasty: periarticular infiltration vs continuous femoral nerve block. British Journal of Anaesthesia. 2010 Aug;105(2):185–95.

16- Andersen LØ, Husted H, Kristensen BB, Otte KS, Gaarn-Larsen L, Kehlet H. Analgesic efficacy of subcutaneous local anaesthetic wound infiltration in bilateral knee arthroplasty: a randomised, placebo-controlled, double-blind trial. Acta Anaesthesiologica Scandinavica. 2010 May;54(5):543–8.

17- Martin F, Martinez V, Mazoit JX, Bouhassira D, Cherif K, Gentili ME, et al. Antiinflammatory Effect of Peripheral Nerve Blocks after Knee Surgery. Anesthesiology. 2008 Sep 1;109(3):484–90.

18- Beswick AD, Dennis J, Gooberman-Hill R, Blom AW, Wylde V. Are perioperative interventions effective in preventing chronic pain after primary total knee replacement? A systematic review. BMJ Open. 2019 Sep;9(9): e028093.

19- Browne C, Copp S, Reden L, Pulido P, Colwell C. Bupivacaine bolus injection versus placebo for pain management following total knee arthroplasty. The Journal of Arthroplasty. 2004 Apr;19(3):377–80.

20- Kim YM, Joo YB, Kang C, Song JH. Can ultrasound-guided nerve block be a useful method of anesthesia for arthroscopic knee surgery? Knee Surg Sports Traumatol Arthrosc. 2015 Jul;23(7):2090–6.

21- Adeyemo WL, Ladeinde AL, Ogunlewe MO. Clinical evaluation of post-extraction site wound healing. J Contemp Dent Pract. 2006 Jul 1;7(3):40–9.

22- von Plato H, Peltoniemi M, Kauhanen P, Löyttyniemi E, Hamunen K, Kontinen V, et al. Combination of perineural and wound infusion after above knee amputation: A randomized, controlled multicenter study. Acta Anaesthesiol Scand. 2019 Nov;63(10):1406–12.

23- Butterfield NN, Schwarz SKW, Ries CR, Franciosi LG, Day B, MacLeod BA. Combined pre- and post-surgical bupivacaine would infiltrations decrease opioid requirements after knee ligament reconstruction. Can J Anesth/J Can Anesth. 2001 Mar;48(3):245–50.

24- Cook P, Stevens J, Gaudron C. Comparing the effects of femoral nerve block versus femoral and sciatic nerve block on pain and opiate consumption after total knee arthroplasty. The Journal of Arthroplasty. 2003 Aug;18(5):583–6.

25- Department of Anaesthesiology and Reanimation, Harran University, School of Medicine, Sanliurfa, Turkey, Altay N, Sarikaya B, Department of Orthopedics and Traumatology, Harran University, School of Medicine, Sanliurfa, Turkey, Karahan MA, Department of Anaesthesiology and Reanimation, Harran University, School of Medicine, Sanliurfa, Turkey, et al. Comparison of efficacy between combined periarticular and incisional injections versus

periarticular injection alone of bupivacaine for pain control after total knee arthroplasty: A prospective randomized controlled trial. AOTT. 2020 Aug 13;54(4):402–7.
26- Kim NY, Lee KY, Bai SJ, Hong JH, Lee J, Park JM, et al. Comparison of the effects of remifentanil-based general anesthesia and popliteal nerve block on postoperative pain and hemodynamic stability in diabetic patients undergoing distal foot amputation: A retrospective observational study. Medicine. 2016 Jul;95(29): e4302.
27-Ludot H, Berger J, Pichenot V, Belouadah M, Madi K, Malinovsky J. Continuous Peripheral Nerve Block for Postoperative Pain Control at Home: A Prospective Feasibility Study in Children. Regional Anesthesia and Pain Medicine. 2008 Jan;33(1):52–6.
28-Ilfeld BM, Moeller LK, Mariano ER, Loland VJ, Stevens-Lapsley JE, Fleisher AS, et al. Continuous Peripheral Nerve Blocks. Anesthesiology. 2010 Feb 1;112(2):347–54.
29-Monahan AM, Madison SJ, Loland VJ, Sztain JF, Bishop ML, Sandhu NS, et al. Continuous Popliteal Sciatic Blocks: Does Varying Perineural Catheter Location Relative to the Sciatic Bifurcation Influence Block Effects? A Dual-Center, Randomized, Subject-Masked, Controlled Clinical Trial. Anesthesia & Analgesia. 2016 May;122(5):1689–95.
30-Lukács J. [Social Welfare Board: safety prescriptions in surgery proposed for Spring 1974]. Tidskr Sver Sjukskot. 1973 Nov 22;40(21):43–4.
31-Grant A, Wood C. The Effect of Intra-Neural Local Anaesthetic Infusion on Pain Following Major Lower Limb Amputation. Scott Med J. 2008 Feb;53(1):4–6.
32- Ilfeld BM, Loland VJ, Gerancher JC, Wadhwa AN, Renehan EM, Sessler DI, et al. The Effects of Varying Local Anesthetic Concentration and Volume on Continuous Popliteal Sciatic Nerve Blocks: A Dual-Center, Randomized, Controlled Study. Anesthesia & Analgesia. 2008 Aug;107(2):701–7.
33-Borgeat A, Blumenthal S, Lambert M, Theodorou P, Vienne P. The Feasibility and Complications of the Continuous Popliteal Nerve Block: A 1001-Case Survey. Anesthesia & Analgesia. 2006 Jul;103(1):229–33.
34-Lai HY, Foo LL, Lim SM, Yong CF, Loh PS, Chaw SH, et al. The hemodynamic and pain impact of peripheral nerve block versus spinal anesthesia in diabetic patients undergoing diabetic foot surgery. Clin Auton Res. 2020 Feb;30(1):53–60.
35-White PF, Issioui T, Skrivanek GD, Early JS, Wakefield C. The Use of a Continuous Popliteal Sciatic Nerve Block After Surgery Involving the Foot and Ankle: Does It Improve the Quality of Recovery? Anesthesia & Analgesia. 2003 Nov;1303–9.
36-Finsen V, Kasseth AM. TOURNIQUETS IN FOREFOOT SURGERY: LESS PAIN WHEN PLACED AT THE ANKLE. The Journal of Bone and Joint Surgery British volume. 1997 Jan;79-B (1):99–101.
37-Li Y, Zhang Q, Wang Y, Yin C, Guo J, Qin S, et al. Ultrasound-guided single popliteal sciatic nerve block is an effective postoperative analgesia strategy for calcaneal fracture: a randomized clinical trial. BMC Musculoskelet Disord. 2021 Dec;22(1):735
38-Rasmussen SB, Saied NN, Bowens C, Mercaldo ND, Schildcrout JS, Malchow RJ. Duration of Upper and Lower Extremity Peripheral Nerve Blockade Is Prolonged with Dexamethasone When Added to Ropivacaine: A Retrospective Database Analysis. Pain Med. 2013 Aug;14(8):1239–47.
39-Zhang AL. Editorial Commentary: The Truth about Peripheral Nerve Blocks and Hip Arthroscopy. Arthroscopy: The Journal of Arthroscopic & Related Surgery. 2019 Sep;35(9):2617–8.
40-Wylde V, Lenguerrand E, Gooberman-Hill R, Beswick AD, Marques E, Noble S, et al. Effect of local anaesthetic infiltration on chronic postsurgical pain after total hip and knee replacement: the APEX randomised controlled trials. Pain. 2015 Jun;156(6):1161–70.

41- Gartke K, Portner O, Taljaard M. Neuropathic Symptoms following Continuous Popliteal Block After Foot and Ankle Surgery. Foot Ankle Int. 2012 Apr;33(4):267–74.
42- Sadoun M, Hardy A, Cladière V, Guichard L, Bauer T, Stiglitz Y. Outpatient total ankle replacement. International Orthopaedics (SICOT). 2021 Sep;45(9):2429–33.
43- Knezevic MM, Stojkovic MZ, Vlajkovic GP, Jovanovic MB, Rasic DM. Pain during external dacryocystorhinostomy with local anesthesia. Med Sci Monit. 2011;17(6):CR341–6.

Reason 3 (n=11) Comparing different types of medication to use for local anaesthetic

1- Spruce MC, Bowling FL, Metcalfe SA. A longitudinal study of hallux valgus surgical outcomes using a validated patient centred outcome measure. The Foot. 2011 Sep;21(3):133–7.
2- Bilgetekin YG, Kuzucu Y, Öztürk A, Yüksel S, Atilla HA, Ersan Ö. The use of the wide-awake local anesthesia no tourniquet technique in foot and ankle injuries. Foot and Ankle Surgery. 2021 Jul;27(5):535–8.
3- Miller SL, Wertheimer SJ. A comparison of the efficacy of injectable dexamethasone sodium phosphate versus placebo in postoperative podiatric analgesia. The Journal of Foot and Ankle Surgery. 1998 May;37(3):223–6.
4- Goldstein RY, Montero N, Jain SK, Egol KA, Tejwani NC. Efficacy of Popliteal Block in Postoperative Pain Control After Ankle Fracture Fixation: A Prospective Randomized Study. Journal of Orthopaedic Trauma. 2012 Oct;26(10):557–61.
5- Zamora FJ, Madduri RP, Philips AA, Miller N, Varghese M. Evaluation of the Efficacy of Liposomal Bupivacaine in Total Joint Arthroplasty. Journal of Pharmacy Practice. 2021 Jun;34(3):403–6.
6- Atanassoff PG, Ocampo CA, Castro Bande M, Hartmannsgruber MWB, Halaszynski TM. Ropivacaine 0.2% and Lidocaine 0.5% for Intravenous Regional Anesthesia in Outpatient Surgery. Anesthesiology. 2001 Sep 1;95(3):627–31.
7- Chan EY, Fransen M, Parker DA, Assam PN, Chua N. Femoral nerve blocks for acute postoperative pain after knee replacement surgery. Cochrane Anaesthesia Group, editor. Cochrane Database of Systematic Reviews [Internet]. 2014 May 13 [cited 2022 May 30]; Available from: https://doi.wiley.com/10.1002/14651858.CD009941.pub2
8- Blichfeldt-Eckhardt MR. From acute to chronic postsurgical pain: the significance of the acute pain response. Dan Med J. 2018 Mar;65(3): B5326.
9- Van Der Zwaard BC, Roerdink RL, Van Hove RP. Increase in early wound leakage in total knee arthroplasty with local infiltrative analgesia (LIA) that includes epinephrine: a retrospective cohort study. Acta Orthopaedica. 2020 Nov 1;91(6):756–60.
10- Horlocker TT, Wedel DJ. Infectious complications of regional anesthesia. Best Practice & Research Clinical Anaesthesiology. 2008 Sep;22(3):451–75.
11- Brattwall M, Turan I, Jakobsson J. Pain Management After Elective Hallux Valgus Surgery: A Prospective Randomized Double-Blind Study Comparing Etoricoxib and Tramadol. Anesthesia & Analgesia. 2010 Aug;111(2):544–9.

Reason 4 (n=12) Assessing outcomes other than pain

1- Anderson JG, Bohay DR, Maskill JD, Gadkari KP, Hearty TM, Braaksma W, et al. Complications After Popliteal Block for Foot and Ankle Surgery. Foot Ankle Int. 2015 Oct;36(10):1138–43.

2- Jeng CL, Torrillo TM, Rosenblatt MA. Complications of peripheral nerve blocks. British Journal of Anaesthesia. 2010 Dec;105: i97–107.

3- Andreae MH, Andreae DA. Regional anaesthesia to prevent chronic pain after surgery: a Cochrane systematic review and meta-analysis †. British Journal of Anaesthesia. 2013 Nov;111(5):711–20.

4- Singelyn FJ. Single-injection applications for foot and ankle surgery. Best Practice & Research Clinical Anaesthesiology. 2002 Jun;16(2):247–54.

5- Saba R, Brovman EY, Kang D, Greenberg P, Kaye AD, Urman RD. A Contemporary Medicolegal Analysis of Injury Related to Peripheral Nerve Blocks. Pain Physician. 2019 Jul;22(4):389–400.

6- Good RP, Snedden MH, Schieber FC, Polachek A. Effects of a preoperative femoral nerve block on pain management and rehabilitation after total knee arthroplasty. Am J Orthop (Belle Mead NJ). 2007 Oct;36(10):554–7.

7- Bugada D, Allegri M, Gemma M, Ambrosoli AL, Gazzerro G, Chiumiento F, et al. Effects of anaesthesia and analgesia on long-term outcome after total knee replacement: A prospective, observational, multicentre study. European Journal of Anaesthesiology. 2017 Oct;34(10):665–72.

8- Womble TN, Comadoll SM, Dugan AJ, Davenport DL, Ali SZ, Srinath A, et al. Is supplemental regional anesthesia associated with more complications and readmissions after ankle fracture surgery in the inpatient and outpatient setting? Foot and Ankle Surgery. 2021 Jul;27(5):581–7.

9- Puljak L, Kojundzic SL, Hogan QH, Sapunar D. Lidocaine Injection into the Rat Dorsal Root Ganglion Causes Neuroinflammation. Anesthesia & Analgesia. 2009 Mar;108(3):1021–6.

10- Sala-Blanch X, López AM, Pomés J, Valls-Sole J, García AI, Hadzic A. No Clinical or Electrophysiologic Evidence of Nerve Injury after Intraneural Injection during Sciatic Popliteal Block. Anesthesiology. 2011 Sep 1;115(3):589–95.

11- Sala-Blanch X, López AM, Pomés J, Valls-Sole J, García AI, Hadzic A. No Clinical or Electrophysiologic Evidence of Nerve Injury after Intraneural Injection during Sciatic Popliteal Block. Anesthesiology. 2011 Sep 1;115(3):589–95.

12- Chelly JE, Delaunay L, Williams B, Borghi B. Outpatient lower extremity infusions. Best Practice & Research Clinical Anaesthesiology. 2002 Jun;16(2):311–20.
